# How experimental biology and ecology can support evidence-based decision-making in conservation: avoiding pitfalls and enabling application

**DOI:** 10.1093/conphys/cox043

**Published:** 2017-08-09

**Authors:** Steven J. Cooke, Kim Birnie-Gauvin, Robert J. Lennox, Jessica J. Taylor, Trina Rytwinski, Jodie L. Rummer, Craig E. Franklin, Joseph R. Bennett, Neal R. Haddaway

**Affiliations:** 1 Canadian Centre for Evidence-Based Conservation and Environmental Management, Department of Biology and Institute of Environmental Science, Carleton University, 1125 Colonel By Drive, Ottawa, Ontario, Canada K1S 5B6; 2 Fish Ecology and Conservation Physiology Laboratory, Department of Biology, Carleton University, 1125 Colonel By Drive, Ottawa, Ontario, Canada K1S 5B6; 3 Australian Research Council (ARC) Centre of Excellence for Coral Reef Studies, James Cook University, Townsville, QLD 4811, Australia; 4 School of Biological Sciences, The University of Queensland, Brisbane, QLD 4072, Australia; 5 EviEM, Stockholm Environment Institute, Box 24218, 10451 Stockholm, Sweden

**Keywords:** Evidence, experimental biology and ecology, relevance, reliability

## Abstract

Policy development and management decisions should be based upon the best available evidence. In recent years, approaches to evidence synthesis, originating in the medical realm (such as systematic reviews), have been applied to conservation to promote evidence-based conservation and environmental management. Systematic reviews involve a critical appraisal of evidence, but studies that lack the necessary rigour (e.g. experimental, technical and analytical aspects) to justify their conclusions are typically excluded from systematic reviews or down-weighted in terms of their influence. One of the strengths of conservation physiology is the reliance on experimental approaches that help to more clearly establish cause-and-effect relationships. Indeed, experimental biology and ecology have much to offer in terms of building the evidence base that is needed to inform policy and management options related to pressing issues such as enacting endangered species recovery plans or evaluating the effectiveness of conservation interventions. Here, we identify a number of pitfalls that can prevent experimental findings from being relevant to conservation or would lead to their exclusion or down-weighting during critical appraisal in a systematic review. We conclude that conservation physiology is well positioned to support evidence-based conservation, provided that experimental designs are robust and that conservation physiologists understand the nuances associated with informing decision-making processes so that they can be more relevant.

## Teaser

Experimental biology and ecology show much promise for informing evidence-based decision making. To do so most immediately and effectively, experimentalists need to consider a number of factors when designing, executing and analyzing experiments to ensure that findings will be deemed relevant and reliable by conservation practitioners.

## Introduction

Humans have dominated the planet for much of the last century (Vitousek *et al.*, 1997), leading to massive environmental change, unprecedented population declines that have triggered the need to designate many organisms as Threatened or Endangered (Mace *et al.*, 2008), and loss of biodiversity (Cardinale *et al.*, 2012; Pimm *et al.*, 2014) to the point now that the current Epoch is referred to as ‘the Anthropocene’ (Crutzen, 2006). The financial resources and government capacity required to make this a ‘good Anthropocene’ (Dalby, 2016) are, however, scarce. We depend on decision-makers to ensure their actions are informed by scientific evidence; yet, it is apparent that environmental practitioners’ decisions are most heavily influenced by past experience, tradition or input from co-workers (Pullin *et al.*, 2004). Practitioners are simply failing to make use of the full body of scientific knowledge, and this can lead to management decisions that fail to achieve the desired outcome, waste precious resources, and/or time (e.g. imperiled populations; Lindenmayer *et al.*, 2013). This series of circumstances has resulted in vast repositories of information being neglected and/or overlooked entirely by decision-makers and practitioners (Cook *et al.*, 2013). The reasons behind this so-called ‘knowledge-action’ gap are numerous and complex (Cook *et al.*, 2013). Literature on the sociology of knowledge reveals that the ways in which practitioners and decision makers consume knowledge and assess knowledge claims is very different than those that typically generate new knowledge (i.e. scientists). Accordingly, scientists and other claimants need to make conscious decisions about whose expectations they hope to meet in their scientific communications and engagement activities and plan their scientific activities accordingly if their aim is to ‘be relevant’ (Young *et al.*, 2016).

Taking the lead from the evidence-based approach that has revolutionized the health sciences (i.e. Cochrane Reviews; see http://www.cochranelibrary.com/cochrane-database-of-systematic-reviews/) and led to standardized practices in the evaluation of evidence, the field of conservation and environmental management is undergoing a paradigm shift toward procedures that provide the critical syntheses needed to properly inform policy and management (Sutherland *et al.*, 2004). One of the largest barriers to evidence-based environmental management is the disconnect between science and policy (Pullin and Knight, 2012). Policy-makers desire a high degree of certainty, which is highly unlikely in science, especially if one views a single empirical study rather than integrating all available evidence. Although traditional approaches to reviewing existing evidence may be familiar to many decision-makers and researchers, such methods are highly susceptible to a suite of biases, including selection bias and publication bias (Sutton *et al.* 2000). Systematic review methods (Pullin and Stewart, 2006; The Cochrane Collaboration, 2011; Collaboration for Environmental Evidence, 2013; Campbell Collaboration, 2016) incorporate steps to reduce the risk of error and bias and have been specifically developed to address the importance of context and the risks of bias in less reliable review methods. Systematic review methods thus aim to ensure access to the best available evidence, yielding more-efficient and less-biased platforms for decision-making than informal reviews (Pullin and Knight, 2009a, b).

Systematic reviews depend on the availability of empirical studies to draw upon as part of the evidence synthesis process. Although scientific studies in the realm of biology and ecology can take many forms, those based on rigorous experimentation are considered to be particularly valuable. In the purest sense, an experiment is a procedure used to verify, refute or validate a hypothesis. What is particularly powerful about rigorous and well-designed experiments is their ability to identify cause-and-effect relationships and response thresholds through careful systematic manipulation of various factors of interest, while controlling against the intrusion of other factors that might otherwise confound the results and interpretation. Experiments can occur in the laboratory or field environment. Other tenets of experimental biology include transparent and repeatable methods, consideration of bias and appropriate use of statistical analyses in a hypothesis-testing framework. Relative to other types of studies (e.g. observational and correlative), experiments provide more certainty with respect to effects. In the context of systematic reviews, a well-designed and executed study is an essential component of evidence synthesis because of its ability to control and describe confounders and sources of heterogeneity. That is, ideally one would assemble a series of independent studies and, often using meta-analytical approaches, aggregate studies to seek more general trends across the broader literature base. Of course, not all experiments are of the highest validity (for a variety of reasons). Therefore, another important part of systematic review is a detailed, critical appraisal of the internal validity (quality) and external validity (generalizability) of each study to determine its utility in the evidence synthesis process.

As systematic reviews become more commonplace in conservation science and environmental management, it is worthwhile to reflect on the characteristics of study design that would be considered indicative of quality. Substantial effort has now been expended on a suite of systematic reviews relating to various conservation and environmental topics. Many of these topics involve studies with classical experimental designs, e.g. biomanipulation for the treatment of eutrophication (Bernes *et al.* 2015). Across these reviews, thousands of studies and experimental designs have been appraised in an attempt to integrate reliable evidence into syntheses to aid decision-making. Our authorship team including those with experience as systematic reviewers and those that identify as experimental biologists, have observed a variety of study designs in experimental biology that can, through differential execution, facilitate or limit the utility of individual studies in syntheses. Here, we summarize some of the most common issues with experimental design that limit relevance to evidence synthesis and management decisions, and propose constructive solutions to improve the utility of experimental biology and ecology research in the increasingly popular field of evidence synthesis.

Increasingly, those working in the realms of biology (e.g. molecular biologists, physiologists and ethologists) and ecology (e.g. community, population and ecosystem ecologists) are conducting research related to understanding our natural world in an applied context. Such research is leading to the development of a number of nascent disciplines (e.g. conservation physiology; Cooke *et al.*, 2013a, the intersection of conservation behaviour and physiology; Cooke *et al.*, 2014a). Even the most fundamental work can be used to support conservation and management activities, directly or indirectly; however, there may be a long lag (years or decades) between knowledge generation and its application or synthesis into synoptic analyses. To assist experimental biologists and ecologists with being ‘relevant’ to conservation and management practitioners and in supporting evidence-based conservation, we provide a perspective on how experimental biology and ecology underpins and promotes evidence-based decision-making in conservation. To do so, we first reflect on the process of evidence synthesis and critical appraisal of study design in systematic reviews. We then present and synthesize key aspects of study quality, referencing ways in which experimental biologists and ecologists can refine their experimental designs and methods to be more likely to be included in systematic reviews. We conclude with a forward-looking perspective on the potential for experimental biology and ecology to revolutionize conservation and management by creating the evidence base that is needed to make informed decisions. We want to be clear that we are not implying that the experimental biology community is doing ‘poor’ science. Rather, the needs and expectations of practitioners can be markedly different than what the ‘typical’ experimental biologist does. Therefore, to ‘be relevant’, it is necessary to consider science through the lens of the potential end users (e.g. stakeholders, managers and policy makers), which will extend the science beyond the scientific community.

## Characteristics of experimental biology and ecology that facilitate evidence-based decisions in conservation

### Cause and effect

Experimental biology and ecology are particularly relevant in the context of evidence-based research, given their focus on identifying causal relationships. The field often integrates experimental, descriptive and theoretical methods to answer study questions, but experimentation is the most powerful tool for determining causality (Werner, 1998). Experimental biology frequently yields quantitative data, which describe mechanisms and processes that can be understood as ‘causally interacting entities’ (Craver, 2007; Weber, 2012). Researchers use model organisms or experimental systems (Weber, 2012) to determine causality by identifying appropriate predictor variables to be manipulated, establishing proper controls, and neutralizing any confounding factors.

A significant benefit to the use of experimental biology in conservation and environmental management is its ability to isolate the effects of stressors (or ‘causes’) by performing experiments under controlled conditions, which allows scientists to separate the effects of a particular variable from other possible effects (Seebacher and Franklin, 2012). This requires a study with two situations: one in which the phenomenon under investigation occurs, and one in which it does not (Weber, 2012). Typically, only a single aspect of the study is altered to establish whether the variation in that aspect is attributed to changes in the response variable. This approach takes advantage of the regularity exhibited by biological mechanisms and assumes that these mechanisms will go from a start point to an end point whenever specific conditions permit (Weber, 2004). Studies of cause and effect are not without their challenges; Nichols *et al.* (2017) suggested that natural variability, multiple stressors, the difficulty of performing rigorous experiments, and the time and money required to undertake such studies collectively make cause and effect studies in field settings somewhat uncommon.

The benefits of increasing power to detect causality in experimental research are especially relevant to the critical appraisal process of systematic review. During critical appraisal, the analyst determines the level of confidence that can be placed on a dataset before incorporating it. In the medical field, a hierarchical approach was proposed to classify the value of data for inclusion in a systematic review (Stevens and Milne, 1997). Similarly, in conservation, the highest level of evidence comes from ‘at least one properly designed, randomized controlled trial of appropriate size’ (Pullin and Knight, 2003). Descriptive studies (e.g. observational and expert opinion) are assigned the lowest ranking in this hierarchy (Concato, 2004) and are likely to be excluded if they do not meet the quality standards set out in the systematic review protocol. Well-designed experiments are needed to generate the evidence necessary to support conservation.

### Ability to explore complex interactions

Biological systems interact with one another in ways that are often difficult to predict (Green and Sadedin, 2005). Experimental biology is relevant to evidence-based science in part due to its ability to explore these complex interactions. The majority of experimental studies isolate and focus on the impacts of a single variable (Blaustein and Kiesecker, 2002). Results of these studies provide an essential foundation to understanding natural systems; however, they are often not representative of natural settings (Christensen *et al.*, 2006). For example, stressors do not often occur in isolation and can interact in complex and unexpected ways (Christensen *et al.*, 2006; Altshuler *et al.*, 2011). Multiple stressors can be additive, synergistic or antagonistic (Folt *et al.*, 1999), making it difficult to formulate adequate unbiased hypotheses and design appropriate studies. Although it may not always be possible to manipulate or isolate variables of interest, experimental biology allows the testing of the combined effects of such stressors, helping to illuminate the nature of these complex interactions (Di Santo, 2015). Although oftentimes challenging to conduct in field settings, experiments that reveal ecological interactions are especially relevant for conservation practitioners (Darling and Côté, 2008; Crain *et al.*, 2009).

It has been argued that the best way to approach the study of complex interactions is through the use of a multi-disciplinary approach (Altshuler *et al.*, 2011) as well as the combination of both holistic and reductionist approaches (Lidicker, 1988). By doing so, researchers integrate genetic, molecular, physiological, ecological and evolutionary approaches for a more complete perspective of the functioning of biological systems. Such approaches have been used to study amphibian population declines (Blaustein and Kiesecker, 2002; van Uitregt *et al.*, 2007), freshwater ecosystem threats (Altshuler *et al.*, 2011), human-induced stressors in marine systems (Crain *et al.*, 2009), and climatic effects on macroalgal recruitment (Lotze and Worm, 2002), among others. These studies have frequently demonstrated complex additive, antagonistic and synergistic effects that will have considerable impacts on the conservation of global ecosystems (Coors and De Meester, 2008). To add to this complexity, ecological carryover effects are also prescribed as crucial considerations in conservation biology. Carryover effects occur in any situation in which an individual’s previous history and experience explains their current performance in a given situation (O’Connor *et al.*, 2014). Failure to assess carryover effects can lead to misguided conclusions and cause further damage to imperiled populations (O’Connor and Cooke, 2015; Ceccato *et al.*, 2016). Recognizing the potential for carryover effects and incorporating that concept into experimental design (e.g. monitoring over longer periods or different life-stages) is important to enhance relevance, particularly in our multiple-stressor world.

## Evidence synthesis in systematic reviews

One of the core elements of a systematic review that differs from other forms of evidence synthesis is the emphasis on critical appraisal of study quality. Although one might assume that papers obtained from the primary peer-reviewed literature are of adequate quality, the reality is that contemporary peer review is not perfect (Smith, 2006). Indeed, if the focus is on where a paper ends up being published rather than if it gets published at all, the process can become distracted (Peres-Neto, 2016). This is particularly problematic, given the rise of predatory journals with little to no peer review (Bartholomew, 2014). In addition, it is generally agreed upon that peer-review itself is a game of chance. So, if a ‘poor quality’ article is submitted enough times, just by chance alone, it is likely to get published (Neff and Olden, 2006). For these reasons, there is dire need for critical appraisal of study internal validity and exclusion of low quality results. Not only does a critique of study quality determine whether a paper may be included or excluded in synthesis, it also can form the basis for differential weighting of studies during meta-analysis (e.g. Detsky *et al.*, 1992). Quoting from the Collaboration for Environmental Evidence (CEE) guidelines ‘study quality assessment requires a number of decisions about the absolute and relative importance of different sources of bias and data quality elements common to environmental data, particularly the appropriateness of temporal and spatial scales. It is therefore vital that the assessment process be standardized and as transparent and repeatable as possible. Quality is a relative term and its measurement and scale are very dependent on the question being addressed. It may be helpful to breakdown the concept of quality into two separate units; study reliability and study relevance’ (Collaboration for Environmental Evidence, 2013).

There are no specific standards for quality assessment when it comes to systematic reviews in the realm of conservation and environmental management. This may not be surprising given that this is also the case for health and medicine, where the use of systematic reviews has a long history (Pullin and Knight, 2001). Bilotta *et al.* (2014) suggested that appraisals of quality must have construct validity, provide consistent results among different reviewers, be broadly applicable, and be easy to implement. However, Katrak *et al.* (2004) concluded that there was no ‘gold standard’ critical appraisal tool for any study design (e.g. experimental, observational, diagnostic and qualitative), nor is there any widely accepted generic tool that can be applied equally well across an array of study types. Of particular interest was, as the authors noted, the fact that there were more critical appraisal methods for experimental than observational study designs (e.g. randomized clinical trials; see Crombie, 1996, e.g. of one critical appraisal approach). The most important critical appraisal elements identified for use in health science by Katrak *et al.* (2004) included random allocation of treatments, appropriateness of outcome measures used, sample size justification/power calculations, study design (whether it was reported) and assessor blinding. Although not all of these are entirely relevant to conservation and management-oriented studies, the concepts are consistent with the aims.

## Pitfalls in experimental approaches when aims are to contribute to evidence-based conservation

### Study reliability

Reliability is the extent to which the design of a given study minimizes susceptibility to bias. There are four primary considerations.

#### Selection bias

Selection bias stems from the manner in which treatment groups (including control groups) are amassed (Kunz and Oxman, 1998). A randomized distribution of experimental units (sites or subjects) to treatments is important to avoid selection bias, though it may not always be feasible. This problem commonly occurs in management situations where control sites are not analogous to treatment sites (i.e. controls are often considered protected areas; Collaboration for Environmental Evidence, 2013). Under these circumstances, temporal comparisons of samples obtained before and after the establishment of protected areas (also known as ‘before-after control-impact’, BACI) is considered the most powerful experimental design (based on considerations of relative inferential strength), and has been successful, e.g. in evaluating the effectiveness of protected areas (Osenberg and Schmitt, 1996; Francini-Filho and Moura, 2008) and road mitigation measures (Rytwinski *et al.*, 2016), and investigating flow regulation services of wetlands (Kadykalo and Findlay, 2016). Concerns of selection bias have been documented for decades (Blackwell and Hodges, 1957), but continue to occur. This form of bias constitutes a major problem for experiments that occur sequentially, because such experimental design does not permit for randomization (Wei, 1978). In some cases, selection bias can be overcome by using a person to select the subjects who is not involved in the experiment itself (Blackwell and Hodges, 1957). However, the most important source of selection bias originates from baseline difference among treatment groups—groups that differ initially cannot reveal treatment differences. This is also known as non-causal association, and can pose a threat to the validity of a study (Lipsitch *et al.*, 2010). Selection bias is not limited to the selection of subjects during an experiment. It can also result from the selection of regions or areas of study, often biased toward easily accessible locations (Phillips *et al.*, 2009). Increased awareness of selection biases and their effects on study reliability can substantially improve experimental reliability. Assigning treatment groups with randomization, stratification or pairing can reduce selection bias, and considering the most relevant approach to a given study can increase the quality of an experiment.

#### Performance bias

Performance bias affects conservation research when treatment groups receive different standards of care, which can affect the outcome of the treatment itself (Collaboration for Environmental Evidence, 2013). In medicine, performance bias arises when subjects known to be in a treatment group are provided a different level of attention or care during follow up, asked more or less detailed questions about symptoms, etc. Any systematic difference that alters the balance of the experiment, the validity of the various treatment groups, and the provision of treatment is performance bias. Performance bias can similarly manifest in ecological studies, e.g. if ambient temperatures are monitored more closely in treatment tanks than in control tanks or if the standard of handling care is different for control group subjects than for those undergoing treatment. Both scenarios could alter the effects of the treatment on the treatment group. When the person collecting the data does so blindly (Kardish *et al.*, 2015), such that they are incapable of differentiating between control and treatment group individuals, performance bias can be mitigated, and the standard of care is more likely to be equivalent. However, blinding is often impractical in ecological experiments, and is therefore, making performance bias difficult to exclude from many studies.

#### Measurement bias

Measurement or detection bias occurs when knowledge of the intervention alters adequate evaluation of the results (Collaboration for Environmental Evidence, 2013). This form of bias is most often addressed through blinding (Schultz *et al.*, 1995), though, again, blinding is often not possible in the context of biology and ecology. It is often impossible to blind researchers to the systems being studied by the very nature of experimental biology often being field-based. However, whenever blinding is possible, it should become standard practice (Philipson and DeSimone, 1997). For example, where manipulative experimental studies alter environmental conditions for an organism (Munday *et al.*, 2016 for good example) in a controlled (e.g. laboratory) or semi-controlled (e.g. mesocosm) environment, measurement bias could be mitigated for by ensuring the individuals taking measurements of organisms are unaware of the treatment applied (e.g. water chemistry differences). Alternatively, where differences between treatment groups are obvious (e.g. water turbidity), where possible sampling and recording can be separated, such that measurement bias can be mitigated during measurement of the target outcome, whilst sampling (e.g. extraction of blood samples, video and photograph recordings) need not be blinded (Hess *et al.*, 2015). Such activities may be done without substantial additional resource requirements.

The use of appropriate technology can also minimize measurement bias by ensuring that the techniques are chosen to best fit subjects and/or species. Some tools used to measure physiological parameters are highly sensitive and should be assessed to ensure they measure the relevant metrics to answer the question at hand. For example, point-of-care devices enable researchers to assess various blood-based physiological parameters of wild animals in remote locations only with careful validation of device performance (Stoot *et al.*, 2014). To answer questions related to ecological parameters, evaluating population growth and other demographics are fundamentally necessary (Freckleton *et al.*, 2006). However, the reliability of population estimates is variable and often depends on the observer (detection bias; Collaboration for Environmental Evidence, 2013) or the population model used, which often has a detection limit that cannot achieve those required to implement changes in many systems (Hovestadt and Nowicki, 2008).

#### Attrition bias

Attrition bias refers to differences in withdrawal rates among groups of a study, leading to incomplete outcome data (Jüni and Egger, 2005). This may result when subjects do not survive and may change the characteristics of the experimental groups (e.g. as has been observed in rodent biomedical research; Holman *et al.*, 2016). Analysts that encounter attrition must consider the reasons for attrition and whether the data are likely to be missing at random or missing due to a reason related to fundamental differences between treatment groups (Harrell, 2015). For example, the researcher may choose to exclude anyone who drops out of an experiment or that disappears from a study area, excluding interesting data that might be related to the treatment (i.e. informative missing). Attrition can therefore compromise randomization (Leon *et al.*, 2006), and may lead to other forms of bias. Attrition is sometimes unavoidable, and remains outside the realm of what researchers can control. Accurate reporting of attrition and justification for the approach used to account for the issue can guide decisions made by systematic reviewers. Nonetheless, the comparison of unequal samples may interfere with the validity of studies (Leon *et al.*, 2006).

### Study relevance

Relevance is often considered in terms of the external validity of the study (The Cochrane Collaboration, 2011); how transferable is it to the context of the question being systematically reviewed? However, appraising study relevance can be more subjective than appraising study quality. Here we discuss four primary considerations.

#### Scale-mismatch

Ecological research is often conducted at the organismal or even down to the sub-organismal scale, but managers typically focus on populations. Exceptions, however, would be for endangered species, for which every individual matters. Therefore, scientists must often extrapolate results to scales that are relevant to management (Cooke *et al.*, 2014b), even if they are not prepared or trained to do so. This has traditionally been a problem when using experimental evidence in conservation (Carpenter, 1996; Fausch *et al.*, 2002), despite the fact that scale-dependency of ecological processes is well known (Levin, 1992; Shea and Chesson, 2002; Holland *et al.*, 2004). However, more experiments are being done at larger temporal and spatial scales (Hautier *et al.*, 2014, 2015). Experimental manipulation is key to these lessons, as is coordination of researchers across experimental sites (Stokstad, 2011).

#### Experimental levels lack relevance

Biologists and ecologists must examine the structure and dynamics of biology from the molecular level, all the way through to populations. Innovative physiological tools available for field studies mean that research is not restricted to the laboratory (Costa and Sinervo, 2004; Stoot *et al.*, 2014). Yet, logistical and time constraints can still limit researchers in their approach to study a particular system or aspect of a system. For example, angling event simulations (Donaldson *et al.*, 2011) or the use of supra-physiological hormone injections (Sopinka *et al.*, 2015) have been widely used in recent years to mimic the natural responses of animals to challenges that they encounter in the wild. These approaches are intended to control treatment variability, but may also lead to a mismatch between study results and true physiological responses (Cooke *et al.*, 2013b). For example, cortisol/corticosterone implants are intended to mimic ‘chronic’ stress in the wild, yet it is unclear the extent to which chronic stress occurs in the wild and what it even looks like in terms of responses (Boonstra, 2013). Indeed, experimental approaches (usually dose-dependent) may be extreme and unrealistic by design, which could make them irrelevant to the natural system. Another common example is laboratory research related to climate change where several static temperatures are selected (e.g. low, medium and high) and organisms are monitored at those temperatures. In most field environments, there is significant thermal heterogeneity in space and time (e.g. diel variation, seasonal fluctuation and thermal refuge); these conditions are difficult to replicate in the laboratory but can be incorporated in experiments (Nay *et al.*, 2015; Habary, 2016). Moreover, if conducting research that is intended to be relevant to thermal aspects of climate change, treatments (i.e. exposure) need to be sufficiently long enough for species to acclimate through phenotypic plasticity and potentially across generations such that epigenetic mechanisms and adaptive responses can be identified (Donelson and Munday, 2015; Donelson *et al.*, 2016; Munday *et al.*, 2016). Consideration of how experimental conditions reflect the experience of wild animals is therefore key to the relevance of studies to systematic review and also to management.

#### Experimental interventions are impractical

Experimental ecology and conservation science can strive to develop solutions that are relevant to the problem and realistic to the end users. Research funding must be directed toward practical solutions that can be acceptable to stakeholders. Co-development of the research agenda among stakeholders (e.g. landowners, public interest groups and recreational/commercial users) is essential to ensuring that solutions considered in experiments could, in addition to being effective, be scaled (i.e. be mass-produced and or quickly and easily implemented) and well-received. To do so, experiments must consider possible economic ramifications of experimental interventions. For example, Cairns *et al.* (2013) studied freshwater turtle bycatch exclusion devices and compared the number of turtles successfully excluded as well as differences in retention of target fish species in nets set by commercial fishers, recognizing that the exclusion devices would only be adopted if they did not interfere with normal operation of the nets or reduce target yields. Similar considerations must be made when assessing the effectiveness of conservation interventions so that resources are allocated to creating actionable interventions that are acceptable to all stakeholders.

#### Use of surrogates

Much conservation and environmental management experimental research aims to understand how environmental alterations will affect species that are already at risk or those living in extreme stenothermal conditions (e.g. Antarctic fishes). These animals are typically very difficult to obtain due to protective laws or expensive and time-consuming retrieval methods, necessitating the use of surrogates (reviewed in Caro and O’Doherty, 1999). Surrogates offer an avenue for experimentation to reduce the cost of some problems (e.g. animal models in medical science; Shanks *et al.*, 2009). In ecology, surrogate species offer an easy alternative (i.e. they are often cultured or raised in captivity) to wild animals; however, surrogates remain different from the species of interest, which can be an important pitfall to experimental science. Studies that fail to consider sex, physiological differences and population variation will provide irrelevant information to management of wild populations. In many scenarios, a single surrogate species is unlikely to satisfy all the criteria for the aim of a study (Lambeck, 1997). In many cases, surrogates are chosen on the basis of historical precedent, charisma, or ease of management (Mealy and Horn, 1981; Sidle and Suring, 1986), and not necessarily for their match to a key species of interest.

Although there are limitations to using surrogate species or populations to develop evidence, they are necessary and useful in many scenarios where it is impossible to attain the necessary sample size for experimentation using the target species/population (Raby *et al.*, 2015). Replication of studies using every possible population of every species is logistically impossible and unnecessary when surrogates can provide relevant information. Understanding when and in what context surrogates are relevant is crucial to conducting high quality research for the many species at risk of extinction. Appropriate consideration of the relationship between the target and surrogate can improve the relevance of such approaches (Wenger, 2008). Differences between target and surrogate species/populations may exist at both genetic and molecular levels as well as at whole animal scales. For example, Ebner *et al.* (2009) developed tag attachment methods on the surrogate *Macquaria ambiguia* for deployment in the closely related but endangered *Macquaria australasica* and found that the surrogate had not provided relevant information. Certainly, conservation triage is useful for identifying the important candidate species and populations for funding and research (Bottrill *et al.*, 2009).

### Repeatability, accuracy and statistical power

#### Insufficient details for repeatability and lack of transparency

Despite the recognized importance of providing sufficient detail for repeatability (Nakagawa and Schielzeth, 2010), lack of such detail is one of the most common pitfalls in experimental biology and ecology. Repeatability is defined as the proportion of the total variance that can be reproduced through repeated measures of the same subjects (Lessells and Boag, 1987), and is often used to evaluate the accuracy, quality and transparency of data, which is of primary concern to researchers (Garamszegi *et al.*, 2009). Unfortunately, the literature in experimental science often uses diffuse and technical calculations to quantify repeatability (Nakagawa and Schielzeth, 2010), which can discourage others to repeat their experiment. A similar pitfall in experimental biology and ecology is the lack of transparency and replicability, though opportunities to maximize transparency have grown considerably with the advance of online publishing (Wicherts *et al.*, 2012) and repositories (e.g. Dryad and FigShare), which facilitates inclusion of raw data, code scripts and other extensive details that support the findings and improves replicability (Roche *et al.*, 2015). It has even been argued that the openness of the scientific realm is what makes it so successful, suggesting that if researchers were more transparent in their way of practicing and publishing science, the fields of biology and ecology would hugely benefit through a reduction of low quality research and gross errors (Wicherts *et al.*, 2012).

An emerging challenge to repeatable science is in the methodology that is essential to reproducing the results of a study. Methodological details are used by systematic reviewers for critical appraisal and quantitative details (e.g. means, variability, sample size and direction of the trend) and are needed for meta-analyses (Haddaway and Verhoeven, 2015). Optimally, field biologists would design studies alongside statisticians with an analytical tool pre-specified to test the hypothesis. Otherwise, bias can be introduced by serial implementation of possible statistical methods until one is identified that yields desired results (p-hacking: Simonsohn *et al.*, 2014; Head *et al.*, 2015). This is not necessarily a devious practice, but one that can arise when the underlying mathematics of statistical models are poorly understood and a belief that models exhibiting the desired results are more likely to be accurate. Furthermore, this can lead to selective reporting of results within a study when researchers fail to report that a statistical test was conducted when the outcome is weak, negative or absent, hindering interpretation of study findings and weakening meta-analytical syntheses (Jennions *et al.*, 2013; Parker *et al.* 2016). Communicating the modelling approach selected by the analysts can support the reliability of the findings. Accurate reporting of exact *P*-values (rather than *P* > 0.05), presenting results for all tested predictor and response variables, and all subgroups of data, listing assumptions tested to validate the statistical method implemented, and citing statistical software and, in some cases, the specific functions or packages used to produce results improves the understanding of the interpretation and allows systematic reviewers to verify the relevance of results when appraising studies for systematic review.

#### Statistical power

A related issue is statistical power (Peterman, 1990; Anderson *et al.*, 2001). In many cases, experimental manipulations are undertaken with a few individuals from a single species to inform management decisions for populations or even multiple species. Particularly with larger animals or species threatened with extinction, population sizes and logistics often do not allow sufficient independent samples to yield robust statistical tests. One potential solution to this issue is relaxing the traditional (and relatively arbitrary) alpha value of 0.05 (Barber and Ogle, 2014; Spanos, 2014). This makes particular sense for issues where the precautionary principle should apply. For example, it may be too costly to sample sufficient numbers of a threatened species to show that an experimental environmental manipulation leads to a ‘significant’ decline using *P* < 0.05. Other researchers have argued for a more ‘information theoretic approach’, which differs from traditional frequentist probability statistics (Burnham and Anderson, 2014). Another solution is the use of larger collaborative, distributed experiments (see ‘Scaling’ above). For example, the approach of coordinated distributed experiments can significantly increase the quality of study designs and boost sample sizes (Fraser *et al.*, 2013) because researchers can more easily pool money for research across a number of projects and plan more comprehensive programmes that achieve superior outcomes.

#### Pseudoreplication

Pseudoreplication occurs when experimental replicates are not statistically independent (Hurlbert, 1984). Pseudoreplication is a controversial issue in ecology, and the subject of considerable debate (Oksanen, 2001; Quinn and Keough, 2002). An unambiguous example is repeat sampling of an individual. For example, in a study investigating how fish respond to varying levels of oxidative stress, repeat samples of the same individual at different stress levels are not independent experimental replicates. A more ambiguous example is an experiment is divided into two tanks, a treatment and control, where 100 fish are measured from each tank. Although the tanks may be the same design, using the same water source and same fish population, there is always a possibility of unmeasured differences that could be responsible for systematic differences between tanks (e.g. proximity to the laboratory door). On the other hand, it is likely impossible to have 100 tanks for each treatment and sample one fish from each tank. Thus, there is often a sacrifice in replication to account for various aspects of experimental design (Oksanen, 2001).

In larger-scale experiments, spatial pseudoreplication can be an additional problem (Hurlbert, 1984). Almost all environmental drivers of biological phenomena are spatially structured. An experimental design that does not account for this spatial structure (e.g. one where many replicates are concentrated in a small portion of the study area, and few in others) can lead to biased effect sizes and statistical significances (Fortin and Dale, 2005; Beale *et al.*, 2010). Likewise, environmental drivers are almost always temporally auto-correlated. Not accounting for this can result in hidden effects that bias results. It must, however, be accepted by experimenters, reviewers and systematic reviewers that all experiments are pseudoreplicated to some extent and that all data rely on some level of interpretation of causality (Oksanen, 2001). Although not all instances of pseudoreplication can be accounted for, there are statistical techniques available to account for lack of independence in some study designs that result in pseudoreplication. Mixed effect models incorporate experimental units in which repeated replicates (e.g. repeated measurements on an individual and multiple samples from a tank) are considered as random effects, each with their own expected error distribution, which can be analyzed alongside the fixed effects of the experimental treatments (Bolker *et al.*, 2007; Zuur *et al.*, 2010). Models incorporating auto-correlation of response variables in space can also be used to alleviate spatial pseudoreplication (Beale *et al.*, 2010). However, there is no substitute for foresight in experimental design to provide the most independent test of experimental treatments as possible (Quinn and Keough, 2002).

### Experimental design and controls

#### Lack of appropriate experimental controls

Control is one of the most basic scientific concepts and is one of the great strengths of experimental approaches to conservation. Incorporating a proper control can provide the power necessary to separate correlation from causation in many studies. Control groups for experimentation may be generated using spatially distinct units, e.g. by comparing sites inside and outside protected areas (Pullin *et al.*, 2013; Twardek *et al.*, 2017, in press) or with temporally distinct units or baselines, such as in BACI designs (Underwood, 1992). Control groups should ideally originate from exactly the same population as the treatment group in a randomized way, but perfect controls may be impossible, particularly in field experiments (Smokorowski and Randall, 2017). Thinking carefully about what appropriate controls are and what factors of the experiment require isolation is necessary to achieving robust results from experimentation. Obtaining baseline physiological values is extremely difficult and potentially impossible for wild animals, particularly where the intervention or impact is already in place when experiments begin. However, there are a variety of techniques recommended for physiological control that involve rapid capture and sampling, e.g. prior to the manifestation of some primary and secondary stress hormones (Pankhurst, 2011). Another example where proper control is difficult is in animal tagging (i.e. biotelemetry; Hussey *et al.*, 2015; Kays *et al.*, 2015). Information about animal behaviour, physiology and ecology in the wild can be used for conservation to inform restoration (Lapointe *et al.*, 2013), combat invasive species (Lennox *et al.*, 2016), or study effects of human impacts on wildlife (Donaldson *et al.*, 2008). All animals must be captured, handled (in some cases anaesthetized), and physically attached to or surgically implanted with a tag to do so. These handling/tagging effects can bias findings by affecting the natural behaviour of animals (Wilson and McMahon, 2006; Jepsen *et al.*, 2015), and control groups are logistically difficult or impossible to obtain in some experimental scenarios (Cooke *et al.* 2013b). Laboratory studies prior to tagging can be used to compare survival and activity of tagged and untagged animals in captivity to determine whether the tagged animals will be representative of the population.

#### Too much control

Control groups for experimentation must be developed with the broader question in mind so that the control and treatment groups in an experiment are established to provide relevant results. Over-control and isolation of too few potential predictor variables limits discovery of the interesting interactions that occur in nature. Systems-based thinking is necessary to establish experiments that can produce relevant, scalable, transferrable results to real world problems in ecology and conservation.

## Conclusion

As a crisis discipline, conservation science strives to develop evidence that is timely, reliable and relevant to management (Soulé, 1985). With technological advancements in remote monitoring of behaviour and physiology, the gap between experimental and field approaches to conservation is narrowing. Novel experimental approaches now possible at larger, more ecologically relevant scales have more power to detect causality in the field and separate complex interactions that exist in nature. Although an expanding human footprint and rapidly changing environment have led to increasingly complex and difficult conservation questions, experimental biology is capable of producing the evidence needed to address many of the key questions so that solutions can be found across a range of relevant scales (Cooke *et al.*, 2014b).

Developing research projects that generate reliable evidence is key but must rely on a solid partnership between scientists, managers and stakeholders. Not only must managers be able to communicate the pressing questions to scientists, they must be able to acknowledge how evidence at various scales can inform policy at the present scale. However, it is generally the responsibility of scientists to consider what scale to work on in a given situation with the available tools. Although the use of experimental biology has permitted great progress in understanding how biological systems interact with one another, we are still far from completely understanding how rapid environmental changes will interact with ecosystem function, population dynamics and individual physiologies (Altshuler *et al.*, 2011). The need to better understand the interactive impacts of multiple stressors is still considered one of the most pressing questions in ecology and conservation (Sala *et al.*, 2000; Crain *et al.*, 2009).

Evidence is increasingly important in managing all aspects of governance, and conservation is becoming one of the leading disciplines in developing protocols and standards for evidence (Dicks *et al.*, 2014). Scientists working in the realm of conservation physiology can have great impacts on conservation (Madliger *et al.*, 2016), but must be aware of the pitfalls outlined above. Educating scientists as to what constitutes the good experimental evidence that is necessary to inform management and policy will be key. In addition, proper reporting protocols for communicating evidence in the literature will be positive for management requiring support for their decision making, scientists striving to produce relevant research, and for the natural systems that all parties must be working collaboratively to manage and conserve.
